# Supplementary material for: The COVID-19 and chloroquine infodemic: Cross-sectional observational study of content analysis on YouTube
Source: PLoS One. 2023 Sep 28;18(9):e0286964. doi: 10.1371/journal.pone.0286964 (PMC10538733; doi:10.1371/journal.pone.0286964)
Supplement: S1 File — (DOCX) [file pone.0286964.s001.docx]

List of analyzed YouTube videos

1. [IHU Méditerranée-Infection](https://www.youtube.com/user/ifr48). (2020, février 28). [*Chloroquine : pourquoi les Chinois se tromperaient-ils ?*](https://www.youtube.com/watch?v=mJl2nPHAo2g) [Vidéo]. YouTube.

2. [IHU Méditerranée-Infection](https://www.youtube.com/user/ifr48). (2020, mars 16). [*Coronavirus : diagnostiquons et traitons ! Premiers résultats pour la chloroquine*](https://www.youtube.com/watch?v=n4J8kydOvbc)*.* [Vidéo]. YouTube.

3. [D Plus Production](https://www.youtube.com/channel/UCTJng3hq9UX-JTbmM6pTUkg). (2020, mars 19). [*President Trump Announces FDA Approval of Hydroxychloroquine for Treatment of Coronavirus*](https://www.youtube.com/watch?v=S1MyHPmSPdg)*.* [Vidéo]. YouTube.

4. [IHU Méditerranée-Infection](https://www.youtube.com/user/ifr48). 2020, mars 24). [*Coronavirus : Remerciements, Toxicité des Traitements, Mortalité*](https://www.youtube.com/watch?v=GFkUnJ46MVI)*.* [Vidéo]. YouTube.

5. [MedCram - Medical Lectures Explained CLEARLY](https://www.youtube.com/user/MEDCRAMvideos). (2020, mars 10). [*Coronavirus Epidemic Update 34: US Cases Surge, Chloroquine & Zinc Treatment Combo, Italy Lockdown*](https://www.youtube.com/watch?v=U7F1cnWup9M)*.* [Vidéo]. YouTube.

6. [Medmastery](https://www.youtube.com/user/MedMastery). (2020, mars 17). [*COVID-19 Update 8: Zinc and chloroquine for the treatment of COVID-19?*](https://www.youtube.com/watch?v=BIymfznD7YA) [Vidéo]. YouTube.

7. [BFMTV](https://www.youtube.com/user/BFMTV). (2020, avril 9). [*Chloroquine: Bernard Tapie défend le Pr Didier Raoult*](https://www.youtube.com/watch?v=9lEwyXEjPAs)*.* [Vidéo]. YouTube.

8. [CNN](https://www.youtube.com/user/CNN). (2020, mars 23). [*Gupta: The truth about using chloroquine to fight coronavirus pandemic*](https://www.youtube.com/watch?v=3AzIgAa0Cm8)*.* [Vidéo]. YouTube.

9. [AsapSCIENCE](https://www.youtube.com/user/AsapSCIENCE). (2020, avril 4). [*Is Hydroxychloroquine The New Coronavirus Cure? | COVID-19 Antivirals*](https://www.youtube.com/watch?v=p0Su-xQHffM)*.* [Vidéo]. YouTube.

10. [Frédérick Moulin](https://www.youtube.com/channel/UC1tFkn2ifypmHfGDLsB7Vpw) (2020, avril 26). [*Chloroquine/Épidémie de coronavirus: itws de Patrick Pelloux, des Prs Didier Raoult/Olivier Bouchaud*](https://www.youtube.com/watch?v=enm5nFJOT48)*.* [Vidéo]. YouTube.

11. [Aurelien Barrau](https://www.youtube.com/channel/UCwgqYNmYaij2_8hq_tOFyZw). (2020, mars 23). [*Covid-19, Chloroquine et crise globale*](https://www.youtube.com/watch?v=5SmNJ0R9ZUg)*.* [Vidéo]. YouTube.

12. [i24NEWS Français](https://www.youtube.com/user/i24newsFR). (2020, mai 14). [*Coronavirus: entretien exclusif avec le professeur Didier Raoult*](https://www.youtube.com/watch?v=yqxvhbciPQ8)*.* [Vidéo]. YouTube.

13. [Gilbert Collard](https://www.youtube.com/user/collardofficiel). (2020, mars 24). [*Gilbert Collard - Podcast : Covid19 et chloroquine ; couple Buzyn-Lévy contre le Professeur Raoult ?*](https://www.youtube.com/watch?v=MA0ilp1fRQg) [Vidéo]. YouTube.

14. [FRANCE 24](https://www.youtube.com/user/france24). (2020, mars 23). [*Chloroquine contre coronavirus : "Je ne vois pas où est l'hésitation"*](https://www.youtube.com/watch?v=wIIX8R5VdPk)*.* [Vidéo]. YouTube.

15. [Washington Post](https://www.youtube.com/user/WashingtonPost). (2020, avril 12). [*Hydroxychloroquine's false hope: How an obscure drug became a coronavirus 'cure' | The Fact Checker*](https://www.youtube.com/watch?v=QC-h7rnZW3k)*.* [Vidéo]. YouTube.

16. [La Ligne Directe Santé](https://www.youtube.com/channel/UCDOwXDzbuZ2BDCfL9gJr_Sg). (2020, mars 26). [*La chloroquine contre le coronavirus ? le cri de révolte d’un médecin français*](https://www.youtube.com/watch?v=Rit3iLxcdFA)*.* [Vidéo]. YouTube.

17. [Dr. Eric Berg DC](https://www.youtube.com/user/drericberg123). (2020, mars 22). [*New Treatment for the Coronavirus (COVID-19): Chloroquine*](https://www.youtube.com/watch?v=9CF_W0XX_Kw)*.* [Vidéo]. YouTube.

18. [Le Parisien](https://www.youtube.com/channel/UCfHn_8-ehdem86fEvlFg-Gw). (2020, mars 24). [*Coronavirus : c’est quoi la chloroquine ?*](https://www.youtube.com/watch?v=j4gBFxoCyzk) [Vidéo]. YouTube.

19. [J'suis pas content TV](https://www.youtube.com/channel/UC9NB2nXjNtRabu3YLPB16Hg). (2020, mars 28). [*CORONAVIRUS: Top 3 des FDP, Cymès mes fesses & Chloroquine autorisée ! (J'SUIS PAS CONTENT! #S06E11)*](https://www.youtube.com/watch?v=0-rBJPrdH7c) [Vidéo]. YouTube.

20. [ABC News](https://www.youtube.com/user/ABCNews). (2020, mars 19). [*Trump touts chloroquine, old malaria drug that doctors say may help treat coronavirus | ABC News*](https://www.youtube.com/watch?v=3GuNbGC2D_8)*.* [Vidéo]. YouTube.

21. [TV5MONDE Info](https://www.youtube.com/channel/UCzKH70qfN_yuXq3s91fdwmg). (2020, mars 23). [*Covid-19 : Chloroquine, le traitement miracle contre le Coronavirus en Suisse ?*](https://www.youtube.com/watch?v=R0Uh0AjWmvY) [Vidéo]. YouTube.

22. [MedCram - Medical Lectures Explained CLEARLY](https://www.youtube.com/user/MEDCRAMvideos). (2020, février 5). [*Coronavirus Epidemic Update 11: Antiviral Drugs, Treatment Trials for nCoV (Remdesivir, Chloroquine)*](https://www.youtube.com/watch?v=pfGpdFNHoqQ)*.* [Vidéo]. YouTube.

23. [Guardian News](https://www.youtube.com/user/guardianwires). (2020, avril 5). [*Trump grilled over continued promotion of hydroxychloroquine to treat coronavirus*](https://www.youtube.com/watch?v=ZTXpRNIDpy0)*.* [Vidéo]. YouTube.

24. [MedCram - Medical Lectures Explained CLEARLY](https://www.youtube.com/user/MEDCRAMvideos). (2020, mars 20). [*Coronavirus Pandemic Update 41: Shelter In Place, FDA Investigates Hydroxychloroquine for COVID-19*](https://www.youtube.com/watch?v=hPz5KxgI_K4)*.* [Vidéo]. YouTube.

25. [Lesnonalignes](https://www.youtube.com/user/lesnonalignes). (2020, mars 25). [*Qui veut empêcher le Pr Raoult et la chloroquine contre le coronavirus ? [Les Non-Alignés]*](https://www.youtube.com/watch?v=LlxvWeZW-38) [Vidéo]. YouTube.

26. [HugoDécrypte](https://www.youtube.com/channel/UCAcAnMF0OrCtUep3Y4M-ZPw). (2020, mars 25). [*Un remède contre le coronavirus trouvé ? La chloroquine de Didier Raoult expliquée simplement*](https://www.youtube.com/watch?v=-nBH1XpNqmA)*.* [Vidéo]. YouTube.

27. [CNEWS](https://www.youtube.com/user/itele). (2020, mars 27). [*Coronavirus : la chloroquine, remède miracle contre l'épidémie ?*](https://www.youtube.com/watch?v=KK9sspYpxI0) [Vidéo]. YouTube.

28. [ABC News](https://www.youtube.com/user/ABCNews). (2020, mars 19). [*FDA approves compassionate use of chloroquine | ABC News*](https://www.youtube.com/watch?v=GKBydiPOcQc)*.* [Vidéo]. YouTube.

29. [Frédérick Moulin](https://www.youtube.com/channel/UC1tFkn2ifypmHfGDLsB7Vpw). (2020, mars 22). [*Chloroquine/IHU–Méditerranée Infection: itw du Pr Éric Chabrière, collaborateur du Pr Didier Raoult*](https://www.youtube.com/watch?v=jp4j-VK7xAM)*.* [Vidéo]. YouTube.

30. [L'info du vrai](https://www.youtube.com/channel/UCpTJXVFYh-r3cSxeQhF7nzg). (2020, mars 27). [*Un élu marseillais traité à la chloroquine contre le virus témoigne*](https://www.youtube.com/watch?v=PTJu9xiztM8)*.* [Vidéo]. YouTube.

31. [RT France](https://www.youtube.com/user/rtenfrancais). (2020, mars 30). [*Chloroquine : témoignage d'une personne atteinte du Covid-19 et traitée à la chloroquine*](https://www.youtube.com/watch?v=7WKBdP07hfk)*.* [Vidéo]. YouTube.

32. [IHU Méditerranée-Infection](https://www.youtube.com/user/ifr48). (2020, mars 3). [*Chloroquine : pourquoi tant de haine ?*](https://www.youtube.com/watch?v=TaV6sj8TuWQ) [Vidéo]. YouTube.

33. [Peak Prosperity](https://www.youtube.com/user/ChrisMartensondotcom). (2020, mars 19). [*Chloroquine: A Promising Coronavirus Treatment?*](https://www.youtube.com/watch?v=rRgnQs6D1u8) [Vidéo]. YouTube.

34. [Radio-Canada Info](https://www.youtube.com/user/RadioCanadaInfo). (2020, mars 20). [*Coronavirus : la chloroquine, un espoir?*](https://www.youtube.com/watch?v=kGr5QrgRYmU) [Vidéo]. YouTube.

35. [Inside Edition](https://www.youtube.com/user/cbstvdinsideedition). (2020, mars 20). [*Coronavirus Patient Tries Drug Used to Treat Malaria*](https://www.youtube.com/watch?v=qtI8xzpSBnA). [Vidéo]. YouTube.

36. [Bloomberg Markets and Finance](https://www.youtube.com/channel/UCIALMKvObZNtJ6AmdCLP7Lg). (2020, mars 19). [*Trump Says Malaria Drug Approved to Treat Coronavirus*](https://www.youtube.com/watch?v=u9NbaXHdfGc)*.* [Vidéo]. YouTube.

37. [ABC News](https://www.youtube.com/user/ABCNews). (2020, mars 20). [*Trump on malaria drug for COVID-19: ‘I feel good about it’*](https://www.youtube.com/watch?v=DNaNX-YsZdI)*.* [Vidéo]. YouTube.

38. [RT France](https://www.youtube.com/user/rtenfrancais). (2020, mai 26). [*Chloroquine: «The Lancet confirme ce que dit le Pr.Raoult, le traitement est inutile en réanimation»*](https://www.youtube.com/watch?v=7k8HoqYtnlc)*.* [Vidéo]. YouTube.

39. [FRANCE 24](https://www.youtube.com/user/france24). (2020, mars 26). [*La chloroquine, une "bonne nouvelle" précipitée*](https://www.youtube.com/watch?v=hYSQg202IGY)*.* [Vidéo]. YouTube.

40. [IHU Méditerranée-Infection](https://www.youtube.com/user/ifr48). (2020, mars 9). [*Coronavirus : Peur vs data / Chloroquine : Recherche clinique*](https://www.youtube.com/watch?v=pb8Q1yr2cjo)*.* [Vidéo]. YouTube.

41. [Sud Radio](https://www.youtube.com/channel/UCESTwDXpoMgiYBHipMdKTkQ). (2020, mars 27). [*Philippe Douste-Blazy - Chloroquine : "Ca fait 50 ans qu'on connaît ce médicament !"*](https://www.youtube.com/watch?v=2EhNs0RGoQ0) [Vidéo]. YouTube.

42. [MANDARIN TV法国华人卫视](https://www.youtube.com/user/viefilmfr). (2020, mars 6). [*Le Pr. Didier RAOULT de l'IHU : la chloroquine un traitement contre le coronavirus ?*](https://www.youtube.com/watch?v=vcYMKwN6u6k) [Vidéo]. YouTube.

43. [FLORIAN BRUCKER](https://www.youtube.com/channel/UC0dyH_ouqwT19cSd-qJJmAA). (2020, mars 28). [*RAOULT DE BERGERAC I // NON MERCI ! #DIDIERRAOULT #CORONAVIRUS #CHLOROQUINE*](https://www.youtube.com/watch?v=mo0LIzdXFkk). [Vidéo]. YouTube.

44. [Union Populaire Républicaine](https://www.youtube.com/user/UPRdiffusion). (2020, mars 31). [*Pourquoi la polémique sur la chloroquine et pas sur les autres médicaments testés par décret ?*](https://www.youtube.com/watch?v=FPKt8fg9eyA) [Vidéo]. YouTube.

45. [TODAY](https://www.youtube.com/user/TODAYNBC). (2020, mars 24). [*Man Dies After Taking Chloroquine Phosphate To Prevent Coronavirus | TODAY*](https://www.youtube.com/watch?v=oq2Q_mV6XUI)*.* [Vidéo]. YouTube.

46. [NBC News](https://www.youtube.com/user/NBCNews). (2020, mars 31). [*FDA authorizes use of antimalarial drugs for coronavirus treatment | NBC Nightly News*](https://www.youtube.com/watch?v=eG6cW97ixAs)*.* [Vidéo]. YouTube.

47. [BBC Afrique](https://www.youtube.com/channel/UCBte7YLdJx-O_YljuvN6whg). (2020, mars 25). [*Chloroquine : Un espoir pour l'Afrique ? BBC Infos - 25/03/2020*](https://www.youtube.com/watch?v=0thS4Dou6_U)*.* [Vidéo]. YouTube.

48. [CNEWS](https://www.youtube.com/user/itele). (2020, mars 23). [*Covid-19 : la chloroquine testée à grande échelle*](https://www.youtube.com/watch?v=2CUx9hbfTqE)*.* [Vidéo]. YouTube.

49. [Gilbert Collard](https://www.youtube.com/user/collardofficiel). (2020, mars 30). [*Gilbert Collard - Podcast : On voudrait rendre la chloroquine inutilisable, on ne ferait pas mieux !*](https://www.youtube.com/watch?v=2KY1C663MYU) [Vidéo]. YouTube.

50. [L'info du vrai](https://www.youtube.com/channel/UCpTJXVFYh-r3cSxeQhF7nzg). (2020, mars 25). [*Essai d'un traitement à la chloroquine par le professeur Didier Raoult pour soigner le virus*](https://www.youtube.com/watch?v=VaClACMW-K0)*.* [Vidéo]. YouTube.

51. [FRANCE 24](https://www.youtube.com/user/france24). (2020, mars 25). [*Pandémie de Covid-19 : Polémique autour de l'utilisation de la chloroquine*](https://www.youtube.com/watch?v=lzuxddzBdRc)*.* [Vidéo]. YouTube.

52. [La Provence](https://www.youtube.com/channel/UC2i-BljAg1w_MMMA1JuFm7w). (2020, février 26). [*Le professeur Didier Raoult explique pourquoi la chloroquine peut traiter facilement le Coronavirus*](https://www.youtube.com/watch?v=fcNRmALkpTA)*.* [Vidéo]. YouTube.

53. [BFMTV](https://www.youtube.com/user/BFMTV). (2020, mars 23). [*Chloroquine, la controverse*](https://www.youtube.com/watch?v=LFNeF8jdCoA)*.* [Vidéo]. YouTube.

54. [La Tronche en Biais](https://www.youtube.com/user/TroncheEnBiais). (2020, avril 9). [*Hydroxychloroquine et COVID-19 : la "méthode" Raoult ne nous aide pas*](https://www.youtube.com/watch?v=rP2PWgnGn24)*.* [Vidéo]. YouTube.

55. [Le Parisien](https://www.youtube.com/channel/UCfHn_8-ehdem86fEvlFg-Gw). (2020, février 26). [*Coronavirus : un traitement contre le paludisme pourrait suffire pour guérir*](https://www.youtube.com/watch?v=7eK-Bt-A0CU)*.* [Vidéo]. YouTube.

56. [ABC7](https://www.youtube.com/user/abc7LosAngeles). (2020, avril 7). [*LA doctor seeing success with hydroxychloroquine to treat COVID-19*](https://www.youtube.com/watch?v=eVs_EWVCVPc)*.* [Vidéo]. YouTube.

57. [Le Monde](https://www.youtube.com/user/LeMonde). (2020, mars 27). [*Hydroxychloroquine contre Covid-19 ? Pourquoi l’étude du Pr. Raoult ne suffit pas pour prescrire*](https://www.youtube.com/watch?v=XwVhWLloXnQ)*.* [Vidéo]. YouTube.

58. [CGTN Français](https://www.youtube.com/user/CCTVFrench). (2020, février 23). [*Ce que l'on sait sur le traitement clinique du coronavirus*](https://www.youtube.com/watch?v=4R0nlfW84I4)*.* [Vidéo]. YouTube.

59. [AJ+ français](https://www.youtube.com/channel/UCd1jQ_ppmKfphnh4p39HMFg). (2020, avril 2). [*CHLOROQUINE : DIDIER RAOULT A-T-IL RAISON ? | RIEN NE VA +*](https://www.youtube.com/watch?v=6E66S7cILLQ)*.* [Vidéo]. YouTube.

60. [Sud Radio](https://www.youtube.com/channel/UCESTwDXpoMgiYBHipMdKTkQ). (2020, avril 3). [*Nicole Delépine -"Il arrêter de dire que la chloroquine c'est dangereux !"*](https://www.youtube.com/watch?v=rO_K-yp6LDQ) [Vidéo]. YouTube.

61. [Public Sénat](https://www.youtube.com/user/publicsenat). (2020, mars 23). [*Chloroquine : le professeur Didier Raoult publie un livre (Revue de presse des territoires)*](https://www.youtube.com/watch?v=j0PMOyN7rJk) [Vidéo]. YouTube.

62. [FRANCE 24](https://www.youtube.com/user/france24). (2020, mars 25). [*La chloroquine comme solution possible face au Covid-19 ?*](https://www.youtube.com/watch?v=MfUvJsypxSw) [Vidéo]. YouTube.

63. [INTELLECT MEDICOS](https://www.youtube.com/channel/UCzq4OEffLlTLHui58ESCGrA). (2020, avril 7). [*COVID-19 UPDATE: HYDROXYCHLOROQUINE | WHEN & HOW TO USE, SIDE EFFECTS, MECHANISM, DOSING !*](https://www.youtube.com/watch?v=poyvh7P-KoM) [Vidéo]. YouTube.

64. [PeopleTV](https://www.youtube.com/user/people). (2020, mars 24). [*Arizona Man Dies After Self-Treating Coronavirus With Chloroquine Phosphate Says Hospital | PeopleTV*](https://www.youtube.com/watch?v=l4vdI4Q1kZg)*.* [Vidéo]. YouTube.

65. [James Hoft](https://www.youtube.com/channel/UCrnm0Enrzik7CfgAhrfmmSQ). (2020, mars 16). [*New Study: Taking Chloroquine Fights Off COVID-19 in 6 Days*](https://www.youtube.com/watch?v=VAV7QkABExw)*.* [Vidéo]. YouTube.

66. [FRANCE 24](https://www.youtube.com/user/france24). (2020, avril 6). [*Coronavirus aux États-Unis : Donald Trump en faveur du traitement à la chloroquine*](https://www.youtube.com/watch?v=wNcNBVGogyc)*.* [Vidéo]. YouTube.

67. [India Today](https://www.youtube.com/channel/UCYPvAwZP8pZhSMW8qs7cVCw). (2020, mars 23). [*Coronavirus: ICMR Prescribes Anti-Malarial Drug Hydroxy-Chloroquine For High-Risk COVID-19 Cases*](https://www.youtube.com/watch?v=6J2voLoMdp8)*.* [Vidéo]. YouTube.

68. [IHU Méditerranée-Infection](https://www.youtube.com/user/ifr48). (2020, mars 17). [*Hydroxychloroquine and azithromycin as a treatment of COVID-19*](https://www.youtube.com/watch?v=ydcrROJFEU0)*.* [Vidéo]. YouTube.

69. [WUSA9](https://www.youtube.com/channel/UCcT6w3xUyVshyR2_2vrMp1w). (2020, mars 18). [*What is Chloroquine and can it cure the Coronavirus? Dr. Ernest Brown's answers*](https://www.youtube.com/watch?v=-3AsQQiHN_g)*.* [Vidéo]. YouTube.

70. [CHRIST TV](https://www.youtube.com/channel/UCgzdwgfCJItgP9Epm0eh02A). (2020, avril 13). [*Coronavirus Chloroquine Prophecy - Prophet Emmanuel Makandiwa*](https://www.youtube.com/watch?v=PygVx9qKfgg)*.* [Vidéo]. YouTube.

71. [FRANCE 24](https://www.youtube.com/user/france24). (2020, mars 27). [*Coronavirus : la prescription du Plaquenil, dérivé de la chloroquine, désormais autorisée*](https://www.youtube.com/watch?v=uMUIAqJCh3s)*.* [Vidéo]. YouTube.

72. [Yourheartdoc](https://www.youtube.com/channel/UCE6NE2tnOAnuOfUBeyBakXg). (2120, mars 22). [*COVID 19 treatment. Do the drugs hydroxychloroquine and azithromycin work for coronarvirus?*](https://www.youtube.com/watch?v=hY5yO1dYkQQ) [Vidéo]. YouTube.

73. [JRE Clips](https://www.youtube.com/channel/UCnxGkOGNMqQEUMvroOWps6Q). (2021, avril 1). [*Is Chloroquine an Effective Treatment for Coronavirus? w/Peter Hotez | Joe Rogan*](https://www.youtube.com/watch?v=uy3cDf_nrwI)*.* [Vidéo]. YouTube.

74. [FRANCE 24](https://www.youtube.com/user/france24). (2020, mars 23). [*Coronavirus - Covid-19 : la chloroquine suscite espoir et controverses*](https://www.youtube.com/watch?v=ftRPlHr928Q)*.* [Vidéo]. YouTube.

75. [CardioGauge](https://www.youtube.com/channel/UC_z1l_iS5ZJFPi2FMHED95g). (2020, mars 23). [*Chloroquine and Hydroxychloroquine for Coronavirus: Does it Work?*](https://www.youtube.com/watch?v=SQHrT_SVcws) [Vidéo]. YouTube.

76. [Europe 1](https://www.youtube.com/user/Europe1). (2020, février 26). [*Traitement contre le Coronavirus : Didier Raoult estime que la chloroquine est "susceptible d'êtr…*](https://www.youtube.com/watch?v=L8YDw3RSy7A) [Vidéo]. YouTube.

77. [Medmastery](https://www.youtube.com/user/MedMastery). (2020, mars 24). [*Hydroxychloroquine and azithromycin for the treatment of COVID-19–Review of study by Didier Raoult*](https://www.youtube.com/watch?v=C8dasnwweuw)*.* [Vidéo]. YouTube.

78. [Vivre sainement](https://www.youtube.com/channel/UCPc4qwIJsi4KPJCtjxdogiQ). (2020, mars 22). [*La chloroquine, le médicament qui dérange beaucoup de monde-PARTIE 1*](https://www.youtube.com/watch?v=wv4xAc2NtJk)*.* [Vidéo]. YouTube.

79. [Jasun Dole](https://www.youtube.com/user/flasponge). (2020, mars 19). [*Chloroquine STUDY 100% CURE RATE Please watch, share and follow*](https://www.youtube.com/watch?v=eInV44ujWDM)*.* [Vidéo]. YouTube.

80. [James Hoft](https://www.youtube.com/channel/UCrnm0Enrzik7CfgAhrfmmSQ). (2020, mars 20). [*HUGE: 3 International Studies Find Chloroquine has 100% Success Rate Treating Coronavirus in 6 Days*](https://www.youtube.com/watch?v=Oy4AJP8nAPg)*.* [Vidéo]. YouTube.

81. [Sud Radio](https://www.youtube.com/channel/UCESTwDXpoMgiYBHipMdKTkQ). (2020, mars 29). [*Valérie Boyer : " L'effet secondaire de la chloroquine est que je vais mieux " - Les Incorrectibles*](https://www.youtube.com/watch?v=E6byo86Da0w)*.* [Vidéo]. YouTube.

82. [Edgy Edge](https://www.youtube.com/channel/UComDuLRUbgX7epF0rpniDKA). (2020, mars 13). [*Coronavirus Treatment Zinc & Chloroquine? Covid-19 Medication | How to Treat Coronavirus? Edgy Edge*](https://www.youtube.com/watch?v=mdKSR_SHZmY)*.* [Vidéo]. YouTube.

83. [ICI Québec](https://www.youtube.com/user/rciciquebec). (2020, mars 23). [*Vaccin, chloroquine et colchicine : la course au traitement contre le coronavirus*](https://www.youtube.com/watch?v=haQWRxMCE7w)*.* [Vidéo]. YouTube.

84. [RTL - On a tellement de choses à se dire](https://www.youtube.com/user/radiortl). (2020, mars 19). [*Coronavirus : le traitement à la chloroquine "ne sera pas miraculeux", prévient un infectiologue sur*](https://www.youtube.com/watch?v=A8TwSzYuo5U)*.* [Vidéo]. YouTube.

85. [The Daily Show with Trevor Noah](https://www.youtube.com/channel/UCwWhs_6x42TyRM4Wstoq8HA). (2020, mai 20). [*Trump Takes Hydroxychloroquine | The Daily Social Distancing Show*](https://www.youtube.com/watch?v=Nd1Qkdt4L2c)*.* [Vidéo]. YouTube.

86. [CNBC Television](https://www.youtube.com/channel/UCrp_UI8XtuYfpiqluWLD7Lw). (2020, mai 18). [*Pres. Donald Trump: I'm taking hydroxychloroquine to prevent coronavirus infection*](https://www.youtube.com/watch?v=7nkwE3didNo)*.* [Vidéo]. YouTube.

87. [DoctorOz](https://www.youtube.com/user/DoctorOz). (2020, avril 8). [*An E.R. Doctor Infected With Covid-19 Back At Work After Using Hydroxychloroquine and Z-Pak Protocol*](https://www.youtube.com/watch?v=lXCpNjl-YyI)*.* [Vidéo]. YouTube.

88. [CNN](https://www.youtube.com/user/CNN). (2020, mai 19). [*Trump says he's taking hydroxychloroquine. Dr. Gupta says he shouldn't*](https://www.youtube.com/watch?v=SVm-ro2Fo9M)*.* [Vidéo]. YouTube.

89. [SciShow](https://www.youtube.com/user/scishow). (2020, avril 11). [*Hydroxychloroquine and COVID-19: What We Know Right Now | SciShow News*](https://www.youtube.com/watch?v=va6j4JITJoE)*.* [Vidéo]. YouTube.

90. [MedCram - Medical Lectures Explained CLEARLY](https://www.youtube.com/user/MEDCRAMvideos). (2020, mars 18). [*Coronavirus Pandemic Update 39: Rapid COVID-19 Spread with Mild or No Symptoms, More on Treatment*](https://www.youtube.com/watch?v=AToF8O5T86s)*.* [Vidéo]. YouTube.

91. [Rudy W. Giuliani](https://www.youtube.com/channel/UC-9J07yyuXQTx_uZQchtwsg). (2020, mars 28). [*EXCELLENT NEWS: Hydroxychloroquine Treatment Effective on 699 Patients*](https://www.youtube.com/watch?v=1TJdjhd_XG8)*.* [Vidéo]. YouTube.

92. [DoctorOz](https://www.youtube.com/user/DoctorOz). (2020, avril 4). [*U.S. Doctor Shares New Results Of How Covid-19 Patients Are Doing On Hydroxychloroquine*](https://www.youtube.com/watch?v=Y3NDMPxTA_k)*.* [Vidéo]. YouTube.

93. [CNN](https://www.youtube.com/user/CNN). (2020, avril 21). [*New study shows hydroxychloroquine didn't work against Covid-19*](https://www.youtube.com/watch?v=uGzBrOCkffw)*.* [Vidéo]. YouTube.

94. [IHU Méditerranée-Infection](https://www.youtube.com/user/ifr48). (2020, mars 17). [*L'hydroxychloroquine et l'azithromycine comme traitement du COVID-19*](https://www.youtube.com/watch?v=SfiUlVpqHfE)*.* [Vidéo]. YouTube.

95. [DoctorOz](https://www.youtube.com/user/DoctorOz). (2020, avril 11). [*Breaking News: The Latest On Hydroxychloroquine*](https://www.youtube.com/watch?v=Kj6ZFpVU0XU)*.* [Vidéo]. YouTube.

96. [FLORIAN PHILIPPOT](https://www.youtube.com/channel/UClaa_CwoQEmSo9Mb_M1f91g). (2020, mai 24). [*Étude anti-hydroxychloroquine et anti-Raoult : DÉSINTOX précis*](https://www.youtube.com/watch?v=FNkMO2kUx28)*.* [Vidéo]. YouTube.

97. [DoctorOz](https://www.youtube.com/user/DoctorOz). (2020, avril 3). [*New Research On Hydroxychloroquine Shows More Positive Signs It Could Be A Treatment For Covid-19*](https://www.youtube.com/watch?v=FN2p17oimhE)*.* [Vidéo]. YouTube.

98. [The Star](https://www.youtube.com/user/thestaronline). (2020, mai 23). [*Health DG: Malaysia has been using hydroxychloroquine to treat Covid-19 patients*](https://www.youtube.com/watch?v=mRCVV27zLxM)*.* [Vidéo]. YouTube.

99. [MedCram - Medical Lectures Explained CLEARLY](https://www.youtube.com/user/MEDCRAMvideos). (2020, avril 23). [*Coronavirus Pandemic Update 60: Hydroxychloroquine Update; NYC Data; How Widespread is COVID-19?*](https://www.youtube.com/watch?v=fn2yk5SbGiw) [Vidéo]. YouTube.

100. [CNEWS](https://www.youtube.com/user/itele). (2020, mai 26). [*Coronavirus : coup d'arrêt pour l'hydroxychloroquine en France*](https://www.youtube.com/watch?v=eiOOQsIMcWk)*.* [Vidéo]. YouTube.

101. [MSNBC](https://www.youtube.com/user/msnbcleanforward). (2020, mai 19). [*Trump Says He's Taking Unproven Drug Hydroxychloroquine | Morning Joe | MSNBC*](https://www.youtube.com/watch?v=AWqRhLUItw0)*.* [Vidéo]. YouTube.

102. [DoctorOz](https://www.youtube.com/user/DoctorOz). (2020, avril 7). [*An Update On Hydroxychloroquine And Z Pak*](https://www.youtube.com/watch?v=Qc0e85wTLbM)*.* [Vidéo]. YouTube.

103. [Peak Prosperity](https://www.youtube.com/user/ChrisMartensondotcom). (2020, avril 22). [*Coronavirus: Debunking The Hydroxychloroquine 'Controversy' (Dr. Chris Martenson)*](https://www.youtube.com/watch?v=dLSYRqcg0wo)*.* [Vidéo]. YouTube.

104. [Seeker](https://www.youtube.com/user/DNewsChannel). (2020, avril 13). [*Here’s the Latest on Hydroxychloroquine and Coronavirus Antivirals*](https://www.youtube.com/watch?v=ZhfHZa5MvBY)*.* [Vidéo]. YouTube.

105. [Dr. Eric Berg DC](https://www.youtube.com/user/drericberg123). (2020, mai 3). [*Hydroxychloroquine versus Remdesivir*](https://www.youtube.com/watch?v=iBma_0oAiMI)*.* [Vidéo]. YouTube.

106. [The Telegraph](https://www.youtube.com/user/telegraphtv). (2020, mai 18). [*Donald Trump says he takes hydroxychloroquine drug for Covid-19*](https://www.youtube.com/watch?v=JsfqJ1TkgP0)*.* [Vidéo]. YouTube.

107. [Amanpour and Company](https://www.youtube.com/channel/UCqqjiYoD0nP1fR6vf5AcSfQ). (2020, mai 12). [*Combating Coronavirus Misinformation: Hydroxychloroquine, Masks and More... | Amanpour and Company*](https://www.youtube.com/watch?v=m8EBHXrum-M)*.* [Vidéo]. YouTube.

108. [ABC News](https://www.youtube.com/user/ABCNews). (2020, mai 18). [*Trump says he’s taking hydroxychloroquine*](https://www.youtube.com/watch?v=1I9Bb8Fbui0)*.* [Vidéo]. YouTube.

109. [Sky News](https://www.youtube.com/user/skynews). (2020, avril 13). [*Is hydroxychloroquine a 'miracle' coronavirus treatment?*](https://www.youtube.com/watch?v=SaPFo4u0ZeQ) [Vidéo]. YouTube.

110. [NBC News](https://www.youtube.com/user/NBCNews). (2020, mai 19). [*President Trump Defends Decision To Take Hydroxychloroquine To Prevent COVID-19 | NBC Nightly News*](https://www.youtube.com/watch?v=MXOHwJyW2Jk)*.* [Vidéo]. YouTube.

111. [MedCram - Medical Lectures Explained CLEARLY](https://www.youtube.com/user/MEDCRAMvideos). (2020, mai 15). [*Coronavirus Pandemic Update 71: New Data on Adding Zinc to Hydroxychloroquine + Azithromycin*](https://www.youtube.com/watch?v=WZq-K1wpur8)*.* [Vidéo]. YouTube.

112. [FRANCE 24](https://www.youtube.com/user/france24). (2020, mai 15). [*Covid-19 : l'hydroxychloroquine, plébiscité par le professeur Didier Raoult, serait inefficace*](https://www.youtube.com/watch?v=iD8-d4CKzuc)*.* [Vidéo]. YouTube.

113. [WPLG Local 10](https://www.youtube.com/channel/UCgVZ0mrM3liHNhRYC5Mchgg). (2020, mai 19). [*Dr. Adam Splaver explains why hydroxychloroquine is now part of COVID-19 treatment*](https://www.youtube.com/watch?v=g0l6PIjudnk)*.* [Vidéo]. YouTube.

114. [Reuters](https://www.youtube.com/user/ReutersVideo). (2020, mai 21). [*UK health workers begin hydroxychloroquine trial*](https://www.youtube.com/watch?v=60yl0ESdST4)*.* [Vidéo]. YouTube.

115. [FiveThirtyEight](https://www.youtube.com/channel/UCXKjhxsfFQUqlNVQzLVnpEA). (2020, avril 7). [*Scientists Don’t Know If Hydroxychloroquine Is Safe For Coronavirus Patients l FiveThirtyEight*](https://www.youtube.com/watch?v=alaew0puXfw)*.* [Vidéo]. YouTube.

116. [Dr. John Campbell](https://www.youtube.com/user/Campbellteaching). (2020, mai 27). [*Hydroxychloroquine Studies*](https://www.youtube.com/watch?v=-7za_j7f3L0)*.* [Vidéo]. YouTube.

117. [One America News Network](https://www.youtube.com/user/1americanews). (2020, avril 5). [*Thousands of doctors agree hydroxychloroquine is best treatment for coronavirus patients*](https://www.youtube.com/watch?v=mrzj0DC-gSc)*.* [Vidéo]. YouTube.

118. [DoctorOz](https://www.youtube.com/user/DoctorOz). (2020, avril 8). [*Why Lupus Patients May Hold The Key To Whether Hydroxychloroquine Could Work - Part 1*](https://www.youtube.com/watch?v=44AniIdSGEU)*.* [Vidéo]. YouTube.

119. [Guardian News](https://www.youtube.com/user/guardianwires). (2020, mai 18). [*Coronavirus: Trump claims he takes hydroxychloroquine despite FDA warnings*](https://www.youtube.com/watch?v=UEYfSuH7Iwk)*.* [Vidéo]. YouTube.

120. [Dr. Yo](https://www.youtube.com/channel/UCqovl57t5x2MtHo5z635KFA). (2020, mars 22). [*Hydroxychloroquine, Chloroquine and Remdesivir : The Coronavirus Cure?*](https://www.youtube.com/watch?v=Zcw29KHJkt0) [Vidéo]. YouTube.

121. [CardioGauge](https://www.youtube.com/channel/UC_z1l_iS5ZJFPi2FMHED95g). (2020, avril 13). [*Biggest Hydroxychloroquine / Azithromycin Study Yet! Part Two of Vive la France!*](https://www.youtube.com/watch?v=jsgGvHUTH6A) [Vidéo]. YouTube.

122. [NowThis News](https://www.youtube.com/user/nowthismedia). (2020, avril 7). [*Trump Suggests False COVID-19 'Treatment' Despite Expert Advice | NowThis*](https://www.youtube.com/watch?v=Ctz1U0V3Y4o)*.* [Vidéo]. YouTube.

123. [CBS News](https://www.youtube.com/user/CBSNewsOnline). (2020, avril 29). [*Trump administration wanted to "flood" states with hydroxychloroquine, Vanity Fair reports*](https://www.youtube.com/watch?v=B9Q7yFGVSKA)*.* [Vidéo]. YouTube.

124. [BBC News](https://www.youtube.com/user/bbcnews). (2020, mai 19). [*Coronavirus: Trump says he is taking unproven drug hydroxychloroquine - BBC News*](https://www.youtube.com/watch?v=zRKQWitAXAc)*.* [Vidéo]. YouTube.

125. [Roland S. Martin](https://www.youtube.com/user/rolandsmartin). (2020, avril 10). [*#HesLying! Trump Says He Is Taking Hydroxychloroquine To Prevent Coronavirus Infection*](https://www.youtube.com/watch?v=hCPU6rYmXrc)*.* [Vidéo]. YouTube.

126. [Glenn Beck](https://www.youtube.com/user/glennbeck). (2020, avril 15). [*HYDROXYCHLOROQUINE: Texas trial on NURSING HOME coronavirus patients finds near PERFECT success rate*](https://www.youtube.com/watch?v=NQYFR5AAPXE)*.* [Vidéo]. YouTube.

127. [FRANCE 24](https://www.youtube.com/user/france24). (2020, mai 26). [*Pandémie de Covid-19 en France : L'hydroxychloroquine fait débat*](https://www.youtube.com/watch?v=01miabe6XL8)*.* [Vidéo]. YouTube.

128. [Sky News](https://www.youtube.com/user/skynews). (2020, mai 21). [*COVID-19: Could hydroxychloroquine really work against coronavirus?*](https://www.youtube.com/watch?v=W7K_bbPT3aE) [Vidéo]. YouTube.

129. [Los Angeles Times](https://www.youtube.com/user/losangelestimes). (2020, mai 19). [*Trump says he’s taking hydroxychloroquine in case he contracts the Coronavirus*](https://www.youtube.com/watch?v=4X4lsfqUauc)*.* [Vidéo]. YouTube.

130. [Dr. Eric Berg DC](https://www.youtube.com/user/drericberg123). (2020, mai 6). [*Hydroxychloroquine Benefits Are Beyond Killing the Virus*](https://www.youtube.com/watch?v=B8HZ0Li5uRI)*.* [Vidéo]. YouTube.

131. [CBS News](https://www.youtube.com/user/CBSNewsOnline). (2020, mai 6). [*Trump says he's taking hydroxychloroquine*](https://www.youtube.com/watch?v=2AjD7GjXDlo)*.* [Vidéo]. YouTube.

132. [ICU REACH](https://www.youtube.com/channel/UCOG2zb1HrbuBhGp4aVd1J2Q). (2020, mars 20). [*COVID 19 and the Use of Hydroxychloroquine*](https://www.youtube.com/watch?v=_Hsu1OU1voQ)*.* [Vidéo]. YouTube.

133. [The View](https://www.youtube.com/user/ABCTheView). (2020, mai 19). [*Rep. Mike Kelly on Pennsylvania Reopening, Hydroxychloroquine and COVID-19 Recovery | The View*](https://www.youtube.com/watch?v=Wx6L4FkKvPU)*.* [Vidéo]. YouTube.

134. [Styxhexenhammer666](https://www.youtube.com/user/Styxhexenhammer666). (2020, avril 18). [*Coronavirus Update: Texas Deploys Hydroxychloroquine, 1.5 Million Active Cases*](https://www.youtube.com/watch?v=CE4afj0zIoo)*.* [Vidéo]. YouTube.

135. [The Telegraph](https://www.youtube.com/user/telegraphtv). (2020, mai 26). [*WHO drops hydroxychloroquine from coronavirus trials after safety fears*](https://www.youtube.com/watch?v=IDKdhMa26Ro)*.* [Vidéo]. YouTube.

136. [Psalm 91](https://www.youtube.com/channel/UC0uTcWL40yFf9l5Vsk6ky6g). (2020, mai 11). [*FYI | Hydroxychloroquine | COVID-19 | MD Speaks Out*](https://www.youtube.com/watch?v=-ByNJtXwFUc)*.* [Vidéo]. YouTube.

137. [JAMA Network](https://www.youtube.com/user/TheJAMANetwork). (2020, mars 28). [*Coronavirus (COVID-19) Update: Chloroquine / Hydroxychloroquine and Azithromycin*](https://www.youtube.com/watch?v=_Ufs5jqWEb0)*.* [Vidéo]. YouTube.

138. [Drbeen Medical Lectures](https://www.youtube.com/user/USMLEOnline). (2020, mars 24). [*COVID-19 Insights: 3 Ways Hydroxychloroquine May Help Fight COVID-19*](https://www.youtube.com/watch?v=yjkPdwlhI8A)*.* [Vidéo]. YouTube.

139. [Late Night with Seth Meyers](https://www.youtube.com/user/LateNightSeth). (2020, mai 19). [*Trump Says He’s Taking Hydroxychloroquine: A Closer Look*](https://www.youtube.com/watch?v=OqPZ42isWbE)*.* [Vidéo]. YouTube.

140. [Fox News](https://www.youtube.com/user/FoxNewsChannel). (2020, avril 22). [*Ingraham: The truth about hydroxychloroquine*](https://www.youtube.com/watch?v=RexUJeWmzSE)*.* [Vidéo]. YouTube.

141. [MedCram - Medical Lectures Explained CLEARLY](https://www.youtube.com/user/MEDCRAMvideos). (2020, mars 12). [*Coronavirus Pandemic Update 35: New Outbreaks & Travel Restrictions, Possible COVID-19 Treatments*](https://www.youtube.com/watch?v=vE4_LsftNKM)*.* [Vidéo]. YouTube.

142. [Fox News](https://www.youtube.com/user/FoxNewsChannel). (2020, mai 19). [*McEnany explains Trump's decision to take hydroxychloroquine*](https://www.youtube.com/watch?v=GpxqtUKaGSQ)*.* [Vidéo]. YouTube.

143. [Fox News](https://www.youtube.com/user/FoxNewsChannel). (2020, mars 31). [*Dr. Oz breaks down most promising COVID-19 treatments*](https://www.youtube.com/watch?v=vQ_mrOkZnbc)*.* [Vidéo]. YouTube.

144. [Fox News](https://www.youtube.com/user/FoxNewsChannel). (2020, avril 5). [*Giuliani: Doctors should decide if we use hydroxychloroquine*](https://www.youtube.com/watch?v=mlqzaIDnWFQ)*.* [Vidéo]. YouTube.

145. [ThePrint](https://www.youtube.com/channel/UCuyRsHZILrU7ZDIAbGASHdA). (2020, mars 21). [*Trump backs & world tests drug cocktail for Covid-19 based on new studies & why this virus so smart*](https://www.youtube.com/watch?v=5AnVv4BQK98)*.* [Vidéo]. YouTube.

146. [Dr. John Campbell](https://www.youtube.com/user/Campbellteaching). (2020, mai 10). [*Does Hydroxychloroquine work?*](https://www.youtube.com/watch?v=1XCP1WzOY6M) [Vidéo]. YouTube.

147. [MSNBC](https://www.youtube.com/user/msnbcleanforward). (2020, mai 18). [*Despite FDA Warnings, Trump Says He's Been Taking Hydroxychloroquine For Weeks | Deadline | MSNBC*](https://www.youtube.com/watch?v=ldg25c6qGn8)*.* [Vidéo]. YouTube.

148. [Sky News Australia](https://www.youtube.com/user/SkyNewsAustralia). (2020, avril 29). [*'No doubt in my mind Hydroxychloriquine is very effective' against COVID-19: Palmer*](https://www.youtube.com/watch?v=NJYSsJN431I)*.* [Vidéo]. YouTube.

149. [Covid19 : Laissons les médecins prescrire](https://www.youtube.com/channel/UCkXoE3-4a9EYVkkc1jE6s1Q). (2020, mai 23). [*Etude Lancet : L'hydroxychloroquine dangereuse ! Vraiment ? Dr. V. Guerin répond sur BFM le 23 mai*](https://www.youtube.com/watch?v=VGi_bG8-vVU)*.* [Vidéo]. YouTube.

150. [CBS This Morning](https://www.youtube.com/user/CBSThisMorning). (2020, mars 26). [*Pharmacists share warning about over-prescription of potential COVID-19 drugs*](https://www.youtube.com/watch?v=3BSvd0WIV20)*.* [Vidéo]. YouTube.

151. [ARIRANG NEWS](https://www.youtube.com/user/arirangnews). (2020, février 13). [*Gov't recommends use of antiviral drugs for COVID-19 treatment*](https://www.youtube.com/watch?v=iEGPtf7QGSM)*.* [Vidéo]. YouTube.

152. [Dr. Eric Berg DC](https://www.youtube.com/user/drericberg123). (2020, avril 12). [*Hydroxychloroquine May NOT Work Without Taking Zinc*](https://www.youtube.com/watch?v=NIpR56oEQhs)*.* [Vidéo]. YouTube.

153. [Larry King](https://www.youtube.com/user/LarryKingNow). (2020, avril 15). [*Dr. Oz sounds off on controversy over anti-malarial drug in COVID-19 battle*](https://www.youtube.com/watch?v=bgrlabcBaHg)*.* [Vidéo]. YouTube.

154. [Nebraska Medicine Nebraska Medical Center](https://www.youtube.com/user/NebraskaMedCenter). (2020, mars 4). [*Clinical trial begins for COVID-19 medication*](https://www.youtube.com/watch?v=pHOXEqhApWw)*.* [Vidéo]. YouTube.

155. [BBC](https://www.youtube.com/user/BBC). (2020, mai 18). [*British GP slams Trump for taking malaria drug to ward off Coronavirus (Covid-19) - BBC*](https://www.youtube.com/watch?v=Wv5BwiFTIV8)*.* [Vidéo]. YouTube.

156. [WWLTV](https://www.youtube.com/channel/UCJCreS7pODbA6XYRsI5fdLg). (2020, mars 19). [*New Orleans area hospitals using hydroxychloroquine*](https://www.youtube.com/watch?v=Jeq2PqywLh0)*.* [Vidéo]. YouTube.

157. [Idriss J. Aberkane](https://www.youtube.com/channel/UCsBPtU4hJkWNQ4kA-IsxgKw). (2020, mars 23). [*Pourquoi RAOULT est un héros ! | IDRISS ABERKANE*](https://www.youtube.com/watch?v=SgxGQ7-3u-I)*.* [Vidéo]. YouTube.

158. [NOP](https://www.youtube.com/user/galaxielle). (2020, mars 22). [*COVID-19 : Le traitement existe ! Le gouvernement est responsable de chaque mort supplémentaire*](https://www.youtube.com/watch?v=-acK_ClEvjE)*.* [Vidéo]. YouTube.

159. [RMC](https://www.youtube.com/user/RMCOFFICIEL). (2020, mars 23). [*Covid-19: RMC a pu joindre un patient contaminé et qui suit le traitement à base de chloroquine*](https://www.youtube.com/watch?v=VOvNltz3ZmQ)*.* [Vidéo]. YouTube.

160. [Silvano Trotta](https://www.youtube.com/channel/UCgJsFMb8rF_aO1qZgvu_2AQ). (2020, mai 23). [*Danger Chloroquine ? Vraiment ?*](https://www.youtube.com/watch?v=KRE6K9QPRpo) [Vidéo]. YouTube.

161. [BFMTV](https://www.youtube.com/user/BFMTV). (2020, février 26). [*La chloroquine, utilisée contre le paludisme, peut-elle aussi combattre le coronavirus ?*](https://www.youtube.com/watch?v=mCO-J90mWfg) [Vidéo]. YouTube.

162. [FRANCE 24](https://www.youtube.com/user/france24). (2020, avril 1). [*Coronavirus au Gabon : ruée sur la chloroquine*](https://www.youtube.com/watch?v=rMMCfaC7z34)*.* [Vidéo]. YouTube.

163. [CNBC Television](https://www.youtube.com/channel/UCrp_UI8XtuYfpiqluWLD7Lw). (2020, mars 23). [*Coronavirus: U.S. to start trials of malaria drug and antibiotic to treat COVID-19*](https://www.youtube.com/watch?v=ZSuicQgnEzg)

164. [The Daily Show with Trevor Noah](https://www.youtube.com/channel/UCwWhs_6x42TyRM4Wstoq8HA). (2020, mars 19). [*Trump’s Bulls**t Corona Cure & Sad Spring Breakers | The Daily Social Distancing Show*](https://www.youtube.com/watch?v=agvHuHugb0k)*.* [Vidéo]. YouTube.

165. [Mr. Sam - Point d'interrogation](https://www.youtube.com/user/SamuelBuisseret). (2020, mars 24). [*COVID19: CE SCIENTIFIQUE, NOTRE SAUVEUR ? • Le Petit Point d'? - 24 mars 2020*](https://www.youtube.com/watch?v=h18tSEYukqE)*.* [Vidéo]. YouTube.

166. [India Today](https://www.youtube.com/channel/UCYPvAwZP8pZhSMW8qs7cVCw). (2020, mars 17). [*Corona-Cure Tracker: After India, US, Australia Sees COVID-19 Patient Recover With HIV/Malaria Drugs*](https://www.youtube.com/watch?v=Wu2Z6bdzlkE)*.* [Vidéo]. YouTube.

167. [Hervé Seitz](https://www.youtube.com/user/HKeyHKey). (2020, mars 26). [*Covid-19 et chloroquine : mensonge et caprice à l'heure d'Internet 2.0*](https://www.youtube.com/watch?v=Bm-GJ4PF9ts)*.* [Vidéo]. YouTube.

168. [DoctorOz](https://www.youtube.com/user/DoctorOz). (2020, mars 23). [*The COVID-19 Treatment Controversy: Interview with the Expert Who Believes He Has a Treatment*](https://www.youtube.com/watch?v=fXEy7Mdyhb0)*.* [Vidéo]. YouTube.
