# Supplementary material for: The COVID-19 and chloroquine infodemic: Cross-sectional observational study of content analysis on YouTube
Source: PLoS One. 2023 Sep 28;18(9):e0286964. doi: 10.1371/journal.pone.0286964 (PMC10538733; doi:10.1371/journal.pone.0286964)
Supplement: S2 File — (DOCX) [file pone.0286964.s002.docx]

**Scoring criteria of the DISCERN score for our study**

We have established our quotation criteria from the website <http://www.discern.org.uk/discern_instrument.php>. We judged the form of the information provided, we did not judge the substance, and in our specific case whether or not chloroquine was suitable for the treatment of COVID-19.

The DISCERN score includes 16 items; each item is graded from 1 (no, quality criterion not completely fulfilled) to 5 (yes, quality criterion completely fulfilled).

1. Are the aims clear?

The publication will be rated higher if the is a clear indication at the beginning of what it is about and what it is mean to cover, what topics are meant to be excluded.

The publication will be rated lower if it does not explain the subject and the purpose of the video at the beginning.

1. Does it achieve its aims?

The publication will be rated higher if the explanations answer the question or the problematic of the item 1.

The publication will be rated lower if it is not relevant to item 1.

1. Is it relevant?

The publication will be rated higher if it addresses the questions that readers might ask and if recommendations and suggestions concerning treatment choices are realistic or appropriate.

The publication will be rated lower if it is off topic, or if it delivers non-factual information with erroneous, discriminatory or racist messages.

1. Is it clear what sources of information were used to compile the publication (other than the author or producer)?

The publication will be rated higher if the main claims or statements made about treatment choices are accompanied by a reference to the sources used as evidence, e.g. a research study or expert opinion. References can be quoted in the video or in the description under the video such as bibliography/reference list or the addresses of the experts or organizations quoted, or external links to the online sources.

The publication will be rated lower if it is based on personal experience only, if the facts cannot be verified.

1. Is it clear when the information used or reported in the publication was produced?

The publication will be rated higher if dates of the main sources of information used to compile the publication and date of any revisions of the publication are clearly identifiable. The date of publication of the YouTube video is always displayed.

The publication will be rated lower if there is only the date of publication of the YouTube video.

1. Is it balanced and unbiased?

The publication will be rated higher if there is a clear indication of whether the publication is written from a personal or objective point of view, and if there is evidence that a range of sources of information was used to compile the publication.

The publication will be rated lower if it focuses on the advantages or disadvantages of one particular treatment choice without reference to other possible choices, if the publication relies primarily on evidence from single cases and if the information is presented in a sensational, emotive or alarmist way.

1. Does it provide details of additional sources of support and information?

The publication will be rated higher if there is suggestions for further reading or for details of other organizations providing advice and information about the condition and treatment choices.

1. Does it refer to areas of uncertainty?

The publication will be rated higher if there is discussion of the gaps in knowledge or differences in expert opinion concerning treatment choices.

The publication will be rated lower if it implies that a treatment choice affects everyone in the same way, e.g. 100% success rate with chloroquine treatment.

1. Does it describe how each treatment works?

The publication will be rated higher if there is a description of how the treatment acts on the body to achieve its effect and if the action mechanisms are detailed.

The publication will be rated lower if it only says that the treatment works without describing its mechanism of action.

1. Does it describe the benefits of each treatment?

The publication will be rated higher if it specifies the benefits as controlling or getting rid of symptoms, preventing recurrence of the condition and eliminating the condition, both short-term and long-term.

1. Does it describe the risks of each treatment?

The publication will be rated higher if it specifies the risks as side-effects, complications and adverse reactions to treatment, both short-term and long-term.

1. Does it describe what would happen if no treatment is used?

The publication will be rated higher if there is a description of the risks and benefits of postponing treatment, of watchful waiting (i.e. monitoring how the condition progresses without treatment) or of permanently forgoing treatment, e.g. the natural evolution of COVID-19.

1. Does it describe how the treatment choices affect overall quality of life?

The publication will be rated higher if it descripts the effects of the treatment choices on day-to-day activity and relationships with family, friends and carers.

The publication will be rated lower if it does not talk about everyday life, and if it focuses only on pathophysiological processes.

1. Is it clear that there may be more than one possible treatment choice?

The publication will be rated higher if there are suggestions of alternatives to consider or investigate further (including choices not fully described in the publication) before deciding whether to select or reject a particular treatment choice.

The publication will be rated lower if it only mentions chloroquine as treatment for COVID-19.

1. Does it provide support for shared decision-making?

The publication will be rated higher if there are suggestions of things to discuss with family, friends, doctors or other health professionals concerning treatment choices, and if it is advisable to be properly informed about the treatment before starting it.

1. Based on the answer to all of the above questions, rate the overall quality of the publication as a source of information about treatment choices. This is an “intuitive summary”, all publications have deficiencies, and after responding the first 15 questions, we developed some feeling for the overall quality of the publication that will help us decide whether it is a useful and appropriate source of information about treatment choices.
