# Supplementary material for: The COVID-19 and chloroquine infodemic: Cross-sectional observational study of content analysis on YouTube
Source: PLoS One. 2023 Sep 28;18(9):e0286964. doi: 10.1371/journal.pone.0286964 (PMC10538733; doi:10.1371/journal.pone.0286964)
Supplement: S3 File — (DOCX) [file pone.0286964.s003.docx]

**Scoring criteria of the modified JAMA score for our study**

The modified JAMA score is made up of 4 core standards to evaluate websites: authorship, attribution, disclosure and currency. Each item scoring from 0 to 1; 0 means quality criterion not completely fulfilled, and 1 means quality criterion completely fulfilled.

1. Authorship: the publication will be scored 1 if authors and contributors, their affiliations and relevant credentials are provided.
2. Attribution: the publication will be scored 1 if references and sources for all content are listed clearly, and all relevant copyright information are noted.
3. Disclosure: the publication will be scored 1 if the web site ownership is prominently and fully disclosed, as should any sponsorship, advertising, underwriting or commercial funding.
4. Currency: the publication will be scored 1 if dates that content was posted and updated is indicated (il fact, the date of publication of the YouTube video is always displayed).
