# Supplementary material for: The COVID-19 and chloroquine infodemic: Cross-sectional observational study of content analysis on YouTube
Source: PLoS One. 2023 Sep 28;18(9):e0286964. doi: 10.1371/journal.pone.0286964 (PMC10538733; doi:10.1371/journal.pone.0286964)
Supplement: Attachment — Submitted filename: Response to Reviewers.docx [file pone.0286964.s005.docx]

Dr Yên-Lan Nguyen

Hôpital Cochin, 27 rue du Faubourg Saint Jacques, 75014 Paris Cedex, France

0033628336324

[Yen-lan.nguyen@aphp.fr](mailto:Yen-lan.nguyen@aphp.fr)

2022, November 09

Dear Reviewers and Academic Editor,

Thank you for your interest in our work. We are pleased to present our revised manuscript.

**Response to Saurav Basu, M.D. Academic Editor:**

**1) You asked us to ensure that our manuscript meets PLOS ONE’s style requirements**.

We ensure that our manuscript meets PLOS ONE’s style requirements, we reworked particularly the tables and figures formatting (we upload our figures files to the PACE) and we renamed our files.

**2)** **You asked us to include additional information about our datas and ensure that we have included a statement specifying whether the collection and analysis method complied with the terms and conditions for the source of the data.**

In our Methods section, we included additional information about our dataset. We specified that the collection and analysis method complied with the conditions of YouTube videos that are publicly available.

**3) You asked us to clarify the sources of funding and state what role the funders took in the study.**

We clarified the source of funding in the cover letter: our study did not require funding for its design, conduct and manuscript preparation, and the authors received no specific funding for this work. Our institution solely covers the cost of publication.

**4) You asked us to specify where the minimal dataset can be found.**

We made the list of analyzed videos (S1_File) and our database available (S1_Table) in the additional files.

**Response to Additional Editor comments:**

**5) You asked us to be more rigorous on the methodology, to explain clearly how we applied the criteria for establishing the quality of the videos using the DISCERN and the modified JAMA scores, and to explain whether there may be biases in the judgement of effectiveness of chloroquine.**

We reinforced our methodology in the manuscript and with several additional files:

- We explained how we applied the criteria of each score in a respective file (S2_File and S3_File) with several concrete examples for each item. The content of these files can be included in the manuscript directly, but this may be a bit overwhelming for the reader.
- We specified in our Methods section that the aim of this work is to evaluate the quality of the information available in YouTube videos and to determine the factors that make a video an appropriate source of information about treatment choices. We did not judge if chloroquine was suitable for the treatment of COVID-19, and the video study was done in June 2020, before the Solidarity study was published.

We hope that we have been able to respond to the points raised during the review process, and we remain at your disposal for any further information.

Sincerely yours,

Dr Cynthia Poncelet and Dr Yên-Lan Nguyen
