# Supplementary material for: The COVID-19 and chloroquine infodemic: Cross-sectional observational study of content analysis on YouTube
Source: PLoS One. 2023 Sep 28;18(9):e0286964. doi: 10.1371/journal.pone.0286964 (PMC10538733; doi:10.1371/journal.pone.0286964)
Supplement: Attachment — Submitted filename: Response to Reviewers.docx [file pone.0286964.s006.docx]

Dr Yên-Lan Nguyen

Hôpital Cochin, 27 rue du Faubourg Saint Jacques, 75014 Paris Cedex, France

0033628336324

[Yen-lan.nguyen@aphp.fr](mailto:Yen-lan.nguyen@aphp.fr)

2023, March 03

Dear Reviewers and Academic Editor,

Thank you for your interest in our work. We are pleased to present our revised manuscript.

**Response to Journal Requirements:**

1) **You asked us to review our reference list to ensure that it is complete and correct.**

We checked all the scientific papers in our references, and none have been retracted.

We checked all the non-scientific articles and web links: indeed, the link in reference 25 was no longer functional and we updated it.

**Response to Reviewer 1:**

2) **You suggested that we complement our study with a more qualitative analysis, including the type of arguments for and against chloroquine in the videos and in the comments.**

We fully agree with you on the important value of a qualitative study on the type of arguments for and against chloroquine.

We wanted to be able to provide a first study reviewing the quality of YouTube video regarding the use of chloroquine for the treatment of COVID-19, based on reproducible quantitative scores already used in the literature.

With these scores, we tried to judge objectively the form of the information provided and if the video is a useful and appropriate source of information about treatment choices; we did not judge the substance, and in our specific case whether or not chloroquine was suitable for the treatment of COVID-19.

3) **You suggested that it would be interesting to know if there are differences in the opinions of men and women regarding the use of chloroquine**.

We carried out this analysis and here is the result (added in the manuscript lines 203-205):

Videos submitted by men were more supportive of chloroquine than videos submitted by women, respectively 64/139 videos (47%) and 5/29 (17%), p = 0,0038 (we removed from this analysis the 2 videos presented by both sexes).

**Response to Reviewer 2:**

4) **You asked us to clarify the temporal analysis.**

We conducted a YouTube search of the 100 most viewed videos on the subject on 4 June. The impact criteria of each video were collected on this single day in order to ensure the transversality of the study (the number of views, likes, dislikes, comments changes very quickly over time). In the video data collected, we looked at the publication dates of the videos. The videos were posted between 4 March and 6 May. The analysis of the evolution of the scores over time was based on the publication dates of the videos.

In the Search Protocol section, we stated that “The impact criteria of each video (number of views, likes, dislikes and comments) were collected on the single day of June 4, 2020, in order to ensure the transversality of the study.” Lines 90, 91.

In the Data Collection section, we stated that “The intrinsic video features collected were the date of publication of the video”, line 97.

In the Statistical Analyses section, we stated that “the evolution of DISCERN and JAMA scores over time, based on the publication date, were analyzed using a Bayesian proportional odds model, considering scores as ordinal outcomes,” lines 135-137.

In the Results section, we stated that “Videos were released between February 5^th^, 2020 and May 27^th^, 2020.” Line 144.

5) **You suggested to clarify the sentence “To our knowledge, we report here the first study reviewing the quality of YouTube video regarding the use of chloroquine for the treatment of COVID-19.” Lines 235-237**

We completed the sentence by specifying “for the English and French languages”.

We hope that we have been able to respond to the points raised during the review process, and we remain at your disposal for any further information.

Sincerely yours,

Dr Cynthia Poncelet and Dr Yên-Lan Nguyen
